# Efficacy and safety of eplerenone treatment for patients with diabetic nephropathy: A meta-analysis

**DOI:** 10.1371/journal.pone.0265642

**Published:** 2022-03-24

**Authors:** Honglei Hu, Xiaodong Zhao, Xingqian Jin, Shujuan Wang, Wenlong Liang, Xiangguo Cong

**Affiliations:** 1 Department of Endocrinology, Zibo Central Hospital. Zibo, China; 2 Department of Endocrinology, The Affiliated Suzhou Hospital of Nanjing Medical University, Suzhou Municipal Hospital, Suzhou, China; Tanta University Faculty of Medicine, EGYPT

## Abstract

Diabetic nephropathy (DN), which is correlated with an increased risk of cardiovascular disease, significantly elevates the morbidity and mortality of patients with diabetes. Recently, the benefits of mineralocorticoid receptor antagonists in chronic kidney disease (CKD), such as their anti-inflammatory and anti-fibrotic properties, have been discovered. Thus, the present meta-analysis aimed to systematically assess the efficacy and safety of eplerenone treatment in patients with DN. Six electronic databases—PubMed, The Cochrane Library, Embase, Web of Science, CNKI (China National Knowledge Infrastructure), and CBM(Chinese BioMedical Literature Database)—were searched to retrieve randomized controlled trials that assessed eplerenone treatment in patients with DN and were published up to July 31, 2021. Eight randomized controlled trials involving 838 patients were included. Between the eplerenone treatment groups and controls, significant differences were identified in 24-h urine protein levels (mean difference [MD], −19.63 [95% CI, −23.73 to −15.53], *P* < 0.00001), microalbuminuria (MD, -7.75 [95% CI, -9.75 to -5.75], *P* < 0.00001), urinary albumin-creatinine ratio (MD, -48.29 [95% CI, -64.45 to -32.14], *P* < 0.00001), systolic blood pressure (SBP) (MD, -2.49 [95% CI, -4.48 to -0.50], *P* = 0.01), serum potassium levels (MD, 0.19 [95% CI, 0.13 to 0.24], *P* < 0.00001), and levels of the renal fibrosis indicator laminin (MD, -8.84 [95% CI, -11.93 to -5.75], *P* < 0.00001). However, for the effect of estimated glomerular filtration rate (MD, 1.74 [95% CI, -0.87 to 4.35], *P* = 0.19) and diastolic blood pressure (MD, -0.51 [95% CI, -1.58 to 0.57], *P* = 0.36), the differences between the two groups were not significant. In addition, no noticeable difference was identified in the adverse events of hyperkalemia and cough between them. These findings suggest that eplerenone exerts beneficial effects on DN by significantly reducing urinary albumin or protein excretion, SBP, and laminin levels, without increasing the incidence of hyperkalemia and other adverse events.

## Introduction

With changes in lifestyle and environmental factors, coupled with the effect of genetic susceptibility, the incidence of type 2 diabetes mellitus (T2DM) has surged worldwide. It is estimated that 7 out of 100 patients will be diagnosed with T2DM by 2030 [1], and approximately 40% of the patients diagnosed with T2DM will also be diagnosed with CKD [2]. As the most common chronic microvascular complication of T2DM, by complying with recent research, diabetic nephropathy (DN) occurs in approximately 50% of patients with diabetes. Besides being a leading cause of end-stage renal disease (ESRD) [3, 4], DN also significantly elevates the risk of cardiovascular disease and all-cause mortality in patients with T2DM [5, 6]. Poor management of DN can seriously reduce the quality of life of patients with T2DM, significantly increase the economic burden, and even shorten life expectancy. The current management of DN consists of the control of cardiovascular risk factors, the reduction of albuminuria, the application of angiotensin-converting enzyme inhibitors (ACEIs)/angiotensin receptor blockers (ARBs), and sodium-glucose cotransporter 2 (SGLT2) inhibitors [7]. According to the results of DAPA-CKD and CREDENCE studies, the application of SGLT-2 inhibitors can effectively improve the compound outcome of renal disease [8, 9], but the risk of CKD progression remains high. In relevant research, eplerenone, as a second-generation mineralocorticoid receptor antagonists (MRAs), exhibits a higher and stronger affinity and can block the harmful effects of excessive activation of MR due to aldosterone mediation (e.g., inflammation and fibrosis [10, 11]). Accordingly, this meta-analysis aimed to systematically assess the efficacy and safety of eplerenone for the treatment of DN.

## Materials and methods

### Search strategy

A systematic search was conducted in six electronic databases (i.e., PubMed, The Cochrane Library, Embase, Web of Science, CNKI, and CBM) until July 31, 2021. All randomized controlled clinical trials (RCTs) assessing the effect of eplerenone alone or together with an ACEI/ARB, compared with placebo for treating DN, were considered eligible.

The search was carried out to identify eligible articles with keywords such as DN, mineralocorticoid receptor antagonists, aldosterone antagonists, and eplerenone.

### Inclusion and exclusion criteria

#### Inclusion criteria

1. *Participants*. (a) Adult male patients and non-pregnant female patients with T2DM and albuminuria diagnosed with DN were included. (b) Study patients with or without a history of mild to moderate hypertension (systolic blood pressure/diastolic blood pressure (SBP/DBP) ≥ 140/90 mmHg), whereas only SBP/DBP ≤ 180/110 mmHg were considered eligible. (c) All randomized controlled trials and randomized crossover studies were included.
2. *Intervention*. This meta-analysis included articles of eplerenone with or without ACEI/ARB administered for at least four weeks to patients with DN.
3. *Outcome measures*. The endpoints were urinary albumin or protein excretion (24-h proteinuria (mg/24h), microalbuminuria (ug/min), and urinary albumin creatinine ratio (UACR, mg/g), estimated glomerular filtration rate (eGFR, ml/min per 1.73 m^2^), SBP (mm/Hg), diastolic blood pressure DBP (mm/Hg), serum potassium levels (mmol/L), and the indicator of renal fibrosis, laminin (LN, ug/L). Adverse effects (e.g., events of hyperkalemia and cough) were also reviewed.

#### Exclusion criteria

(1) Duplicate published or incomplete articles; (2) meta-analyses, case reports, letters, and meeting abstracts; (3) articles without the full text and the required outcome indicators; (4) nonhuman clinical trials and non-RCTs of eplerenone therapy; (5) articles of DN with an unclear diagnosis or combined with other diseases.

### Study selection and data extraction

By complying with the inclusion and exclusion criteria, two authors screened the titles and abstracts of the respective articles and read the full text of the articles that met the criteria. Finally, the data were independently extracted. Any disagreement between the two authors was addressed by consensus or by a third investigator. The data collected were the names of the first authors, publication years, and countries studied in the articles, the study design, and the number of patients involved, interventions, study duration, and endpoints of the study.

### Assessment of risk of bias

Assessment of risk of bias was conducted by the two authors using the Cochrane Collaboration’s risk-of-bias assessment tool, which covered the following: (1) random sequence generation (selection bias); (2) allocation concealment (selection bias); (3) blinding of participants and personnel (performance bias); (4) blinding of outcome assessment (detection bias); (5) incomplete outcome data (attrition bias); (6) selective reporting (reporting bias); and (7) other bias.

### Statistical analysis

RevMan 5.3 (The Cochrane Collaboration, UK) software was used for the meta-analysis. Continuous variables were analyzed using the mean difference (MD) and 95% confidence interval (CI), and dichotomous variables were expressed as relative risks (RRs) with 95% confidence interval (CI). The *I*^*2*^ statistic was used to assess statistical heterogeneity, which was not considered significant when *I*^*2*^ < 50% and *P* > 0.1, and the fixed-effect model analysis was conducted to pool the data. While *I*^*2*^ exceeded 50% and *P* < 0.1, substantial heterogeneity existed, and the random effect model was selected. Sensitivity analyses were conducted to assess the contribution of individual trials to the pooled effect estimates by sequentially omitting the respective trials. The graphic data were presented through forest plots, and funnel plots were used to test for publication bias.

## Results

### Search results

**Fig 1:**
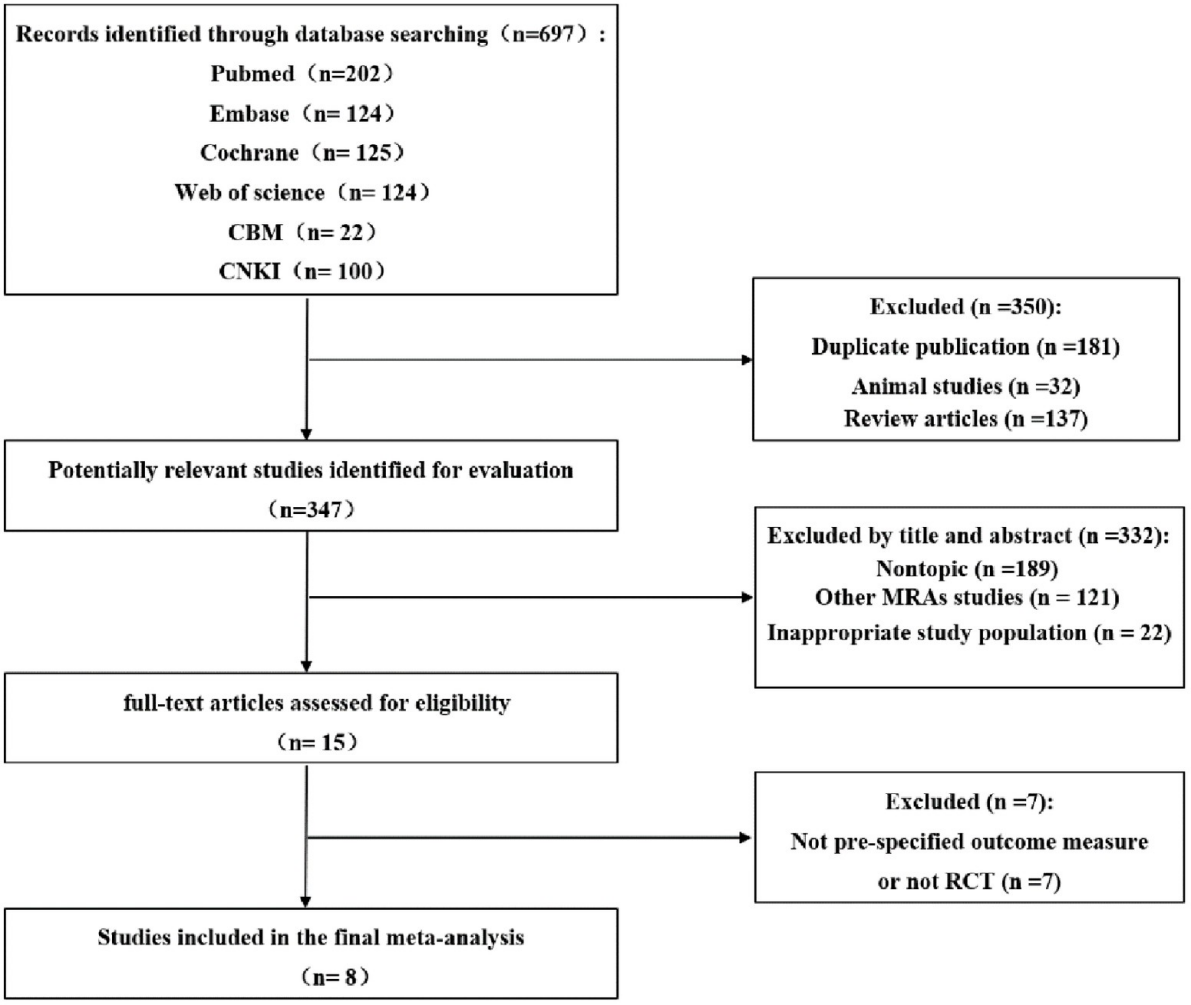
Flow chart of the article search process.

After the article retrieval, a total of 697 eligible articles were obtained, and eight articles were selected for the present meta-analysis [12–19], excluding duplicate publications (181), animal articles (32), reviews (137), nonrelevant study objectives (189), other MRA articles (121), inappropriate study populations (22), and outcome measures that were not pre-specified (7). The study selection process is depicted as a flow diagram in Fig 1.

### Characteristics of eligible articles and quality assessment

**Fig 2:**
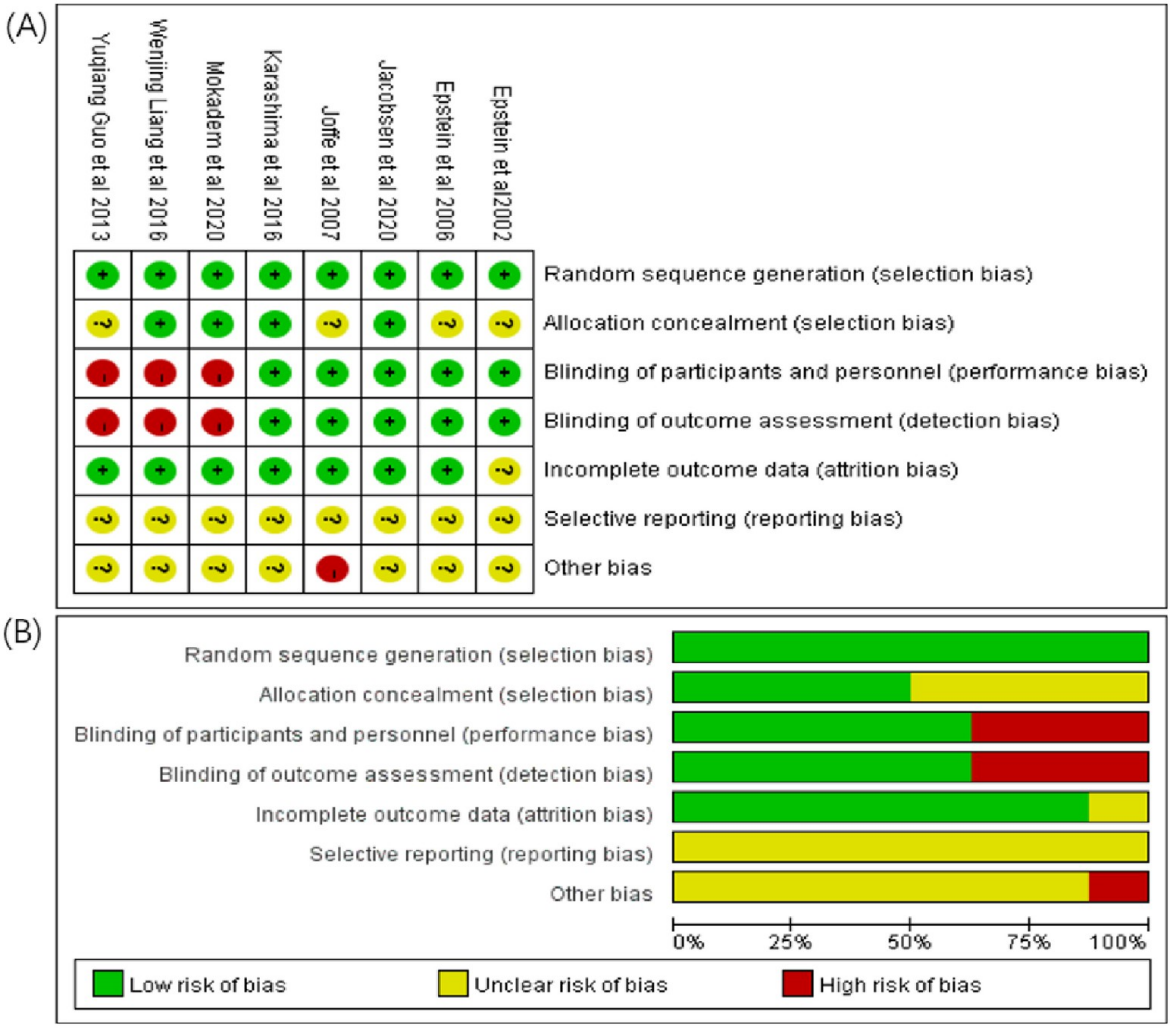
The risks of bias. (A) Risk of bias summary: review authors judgements about the respective risk of bias item for the respective included study. (B)Risk of bias graph: review researchers’ judgements about the respective risk of bias item presented as percentages across all included articles.

The final eligible articles were eight randomized controlled trials, with 838 participants (444 in the treatment group and 394 in the control group), published from 2002 to 2020. The characteristics of the component trials and study patients are shown in Table 1. The risks of bias in the selected eligible articles are shown in Fig 2.

**Table 1:** The characteristics of the included observational articles.

No	Author	Year	Country	Study design	No. of patients Included (Male/Female)	Interventions	Study duration	Endpoints
1	Epstein et al	2006	USA	Parallel RCT	T: 177 (116/61)	T: EPL + ACEI	12 weeks	BP, UACR, eGFR, potassium
					C: 91 (50/41)	C: placebo + ACEI		
2	Joffe et al	2007	USA	Cross-over design	T:8	T: EPL + ACEI	6 weeks	BP, 24h-proteinuria, creatinine, estimated creatinine clearance, potassium
						C: HCTZ + potassium + ACEI		
					C:8			
3	Yuqiang Guo	2013	China	Parallel RCT	T:30	T: EPL	12 weeks	BP, albuminuria, 24h-proteinuria, potassium, LN, PCIII, CIV
					C:60	C: Placebo/SPR		
4	Wenjing Liang et al	2016	China	Parallel RCT	T:42(19/23)	T: EPL	12 weeks	Albuminuria, 24h-proteinuria, LN, PCIII, CIV
					C:41(22/19)	C: SPR		
5	Jacobsen et al	2020	Denmark	Parallel RCT	T: 70 (53/17)	T: EPL	26 weeks	BP, UACR, eGFR, potassium
					C: 70 (53/17)	C: placebo		
6	Mokadem et al	2020	Egypt	Parallel RCT	T: 25 (15/10)	T: EPL + ACEI	24 weeks	BP, UACR, eGFR, creatinine, potassium
					C: 25 (13/12)	C: ACEI		
7	Karashima et al	2016	Japan	Parallel RCT	T: 25 (17/8)	T: EPL + ARB	48 weeks	BP, UACR, eGFR, potassium
					C: 25 (17/8)	C: HCTZ + ARB		
8	Epstein et al	2002	USA	Parallel RCT	T: 67	T: EPL + ACEI	24 weeks	BP, UACR
					C: 74	C: ACEI		

Abbreviations: T, treatment group; C, control group; RCT, randomized controlled trial; EPL, eplerenone; ACEI, angiotensin converting enzyme inhibitors; UACR, urinary albumin-creatinine ratio; eGFR, estimated glomerular filtration rate; BP, blood pressure; SPR, spironolactone; HCTZ, Hydrochlorothiazide.

Among the eight articles selected, random sequence generation was clear (100%), and four articles (50%) were assigned with adequate hiding, while the other four articles (50%) were assigned with unclear hiding. For blinding design, five articles (62.5%) were suggested to be double-blind, one (12.5%) was single-blind, and two articles (25%) were non-blind. Among the eight articles, seven (87.5%) had clear results and one study lacked partial data. Except for one study, which had other biases due to the small sample size, the other articles were unclear about the bias of selective reporting and other biases. In addition, the analysis showed that publication bias was not significant in the selected articles.

### Efficacy outcomes

#### Effect of eplerenone treatment on urinary albumin or protein excretion

There was a significant difference in urinary albumin or protein excretion (24-h proteinuria, microalbuminuria, or UACR) between the eplerenone treatment groups and the controls. The selected articles were categorized into two subgroups which using different controls: hydrochlorothiazide (HCTZ)/spironolactone (SPR), and placebo. The results of the meta-analysis are as follows:

#### Effect of eplerenone treatment on 24-h proteinuria

**Fig 3:**
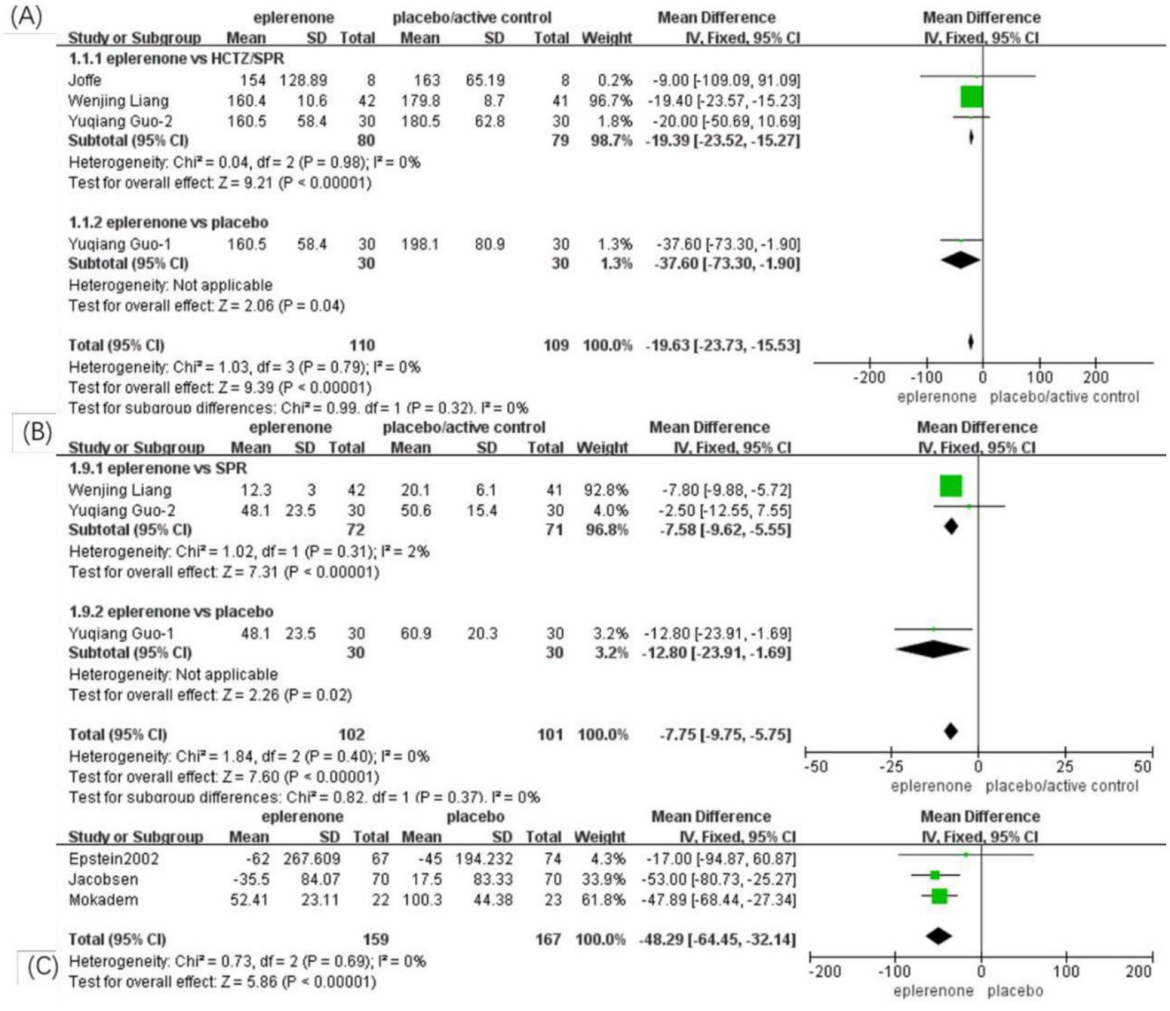
Forest plots of the effects on urinary albumin or protein excretion between the eplerenone treatment groups and the controls. (A) Effect on 24-h Proteinuria in articles comparing eplerenone to placebo/active control. (B) Effect on microalbuminuria in articles comparing eplerenone to placebo/active control. (C) Effect on UACR for the eplerenone versus placebo.

Based on the above subgroup analysis, four articles (n = 219) showed no significant heterogeneity between the articles in the eplerenone subgroup vs. HCTZ/SPR (*Chi*^*2*^ = 0.04, *P* = 0.98, *I*^*2*^ = 0%), the fixed effects model was adopted. A significant reduction was identified in the two subgroups, eplerenone vs. HCTZ/SPR (MD, -19.39 [95% CI, -23.52 to -15.27], *P* < 0.00001) and eplerenone vs. placebo (MD, -37.60 [95% CI, -73.30 to -1.90], *P* < 0.04). In addition, no significant heterogeneity was identified between the two subgroups (*Chi*^*2*^ = 0.99, *P* = 0.32, *I*^*2*^ = 0%). Based on all of the above analyses, the total effect is that the 24-h proteinuria reduced significantly in the eplerenone treatment groups than in the controls (MD, -19.63 [95% CI, -23.73 to -15.53], *P* < 0.00001) (Fig 3A).

#### Effect of eplerenone treatment on microalbuminuria

No significant heterogeneity was identified (three articles, n = 203, *Chi*^*2*^ = 1.02, *P* = 0.31, *I*^*2*^ = 2%), and the fixed effects model was adopted. A significant reduction in microalbuminuria was identified in the eplerenone vs. SPR subgroup (MD, -7.58 [95% CI, -9.62 to -5.55], *P* < 0.00001) and the eplerenone vs. placebo subgroup (MD, —12.80 [95% CI, 23.91 to -1.69], *P* = .02). In addition, no significant heterogeneity was identified between the two subgroups (*Chi*^*2*^ = 0.82, *P* = 0.37, *I*^*2*^ = 0%). The total effect is that microalbuminuria reduced significantly in the eplerenone treatment groups than in the controls (MD, -7.75 [95% CI, -9.75 to -5.75], *P* < 0.00001) (Fig 3B).

#### Effect of eplerenone treatment on UACR

Three articles (n = 326) showed a significant decline between the eplerenone treatment groups and the controls. The fixed effects model was adopted because no significant heterogeneity was identified between the articles analyzed (*Chi*^*2*^ = 0.73, *P* = 0.69, *I*^*2*^ = 0%). A significant reduction in UACR was observed in the eplerenone treatment groups compared with the controls (MD, -48.29 [95% CI, 64.45 to -32.14], *P* < 0.00001) (Fig 3C).

#### Effect of eplerenone treatment on eGFR

**Fig 4:**
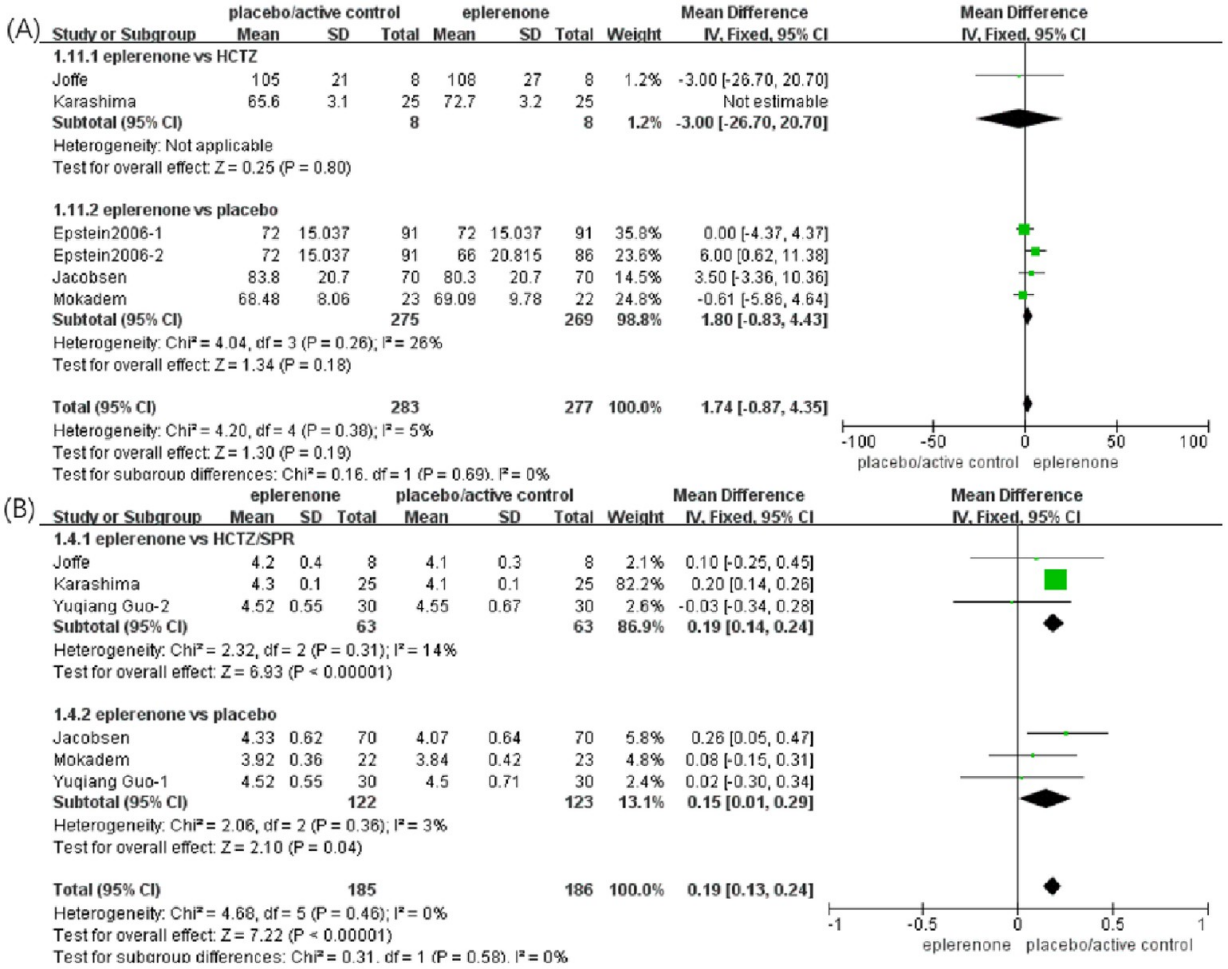
Forest plots of the effects between the eplerenone treatment groups and the controls. (A) Effect on eGFR in articles comparing eplerenone to placebo/active control. (B) Effect on serum potassium levels in articles comparing eplerenone to placebo/active control.

Six articles (n = 610) showed changes in eGFR between the eplerenone treatment groups and the controls. After heterogeneity analysis, one study with high heterogeneity was excluded from further analysis, and the remaining articles had no significant heterogeneity between the two subgroups (*Chi*^*2*^ = 0.16, *P* = 0.69, *I*^*2*^ = 0%). In one study comparing eplerenone to HCTZ, no significant change was indicated (MD, -3 [95% CI, -26.7 to -20.7], *P* < 0.80), and in four articles comparing eplerenone to placebo, no significant difference was identified either (MD, 1.80 [95% CI, -0.80 to 4.43], *P* = 0.18). In a pooled analysis of all six articles, no significant effect on eGFR was identified in the eplerenone treatment group compared with the control group (MD, 1.74 [95% CI, -0.87 to 4.35], *P* = 0.19) (Fig 4A).

#### Effect of eplerenone treatment on serum potassium levels

Six articles (n = 371) showed obvious differences in potassium levels between the eplerenone treatment groups and the controls (MD, 0.19 [95% CI, 0.13 to 0.24], *P* = 0.00001). The selected articles were divided into two subgroups using different controls. In three articles comparing eplerenone to HCTZ/SPR, an increase in serum potassium levels (MD, 0.19 [95% CI, 0.14 to 0.24], *P* = 0.0001) was indicated, and in other three articles comparing eplerenone to placebo, a lower increase was identified (MD, 0.15 [95% CI, 0.01 to 0.29], *P* = 0.04) (Fig 4B).

#### Effect of eplerenone treatment on blood pressure

The effect of eplerenone treatment on blood pressure was assessed in six articles (n = 371).

**Fig 5:**
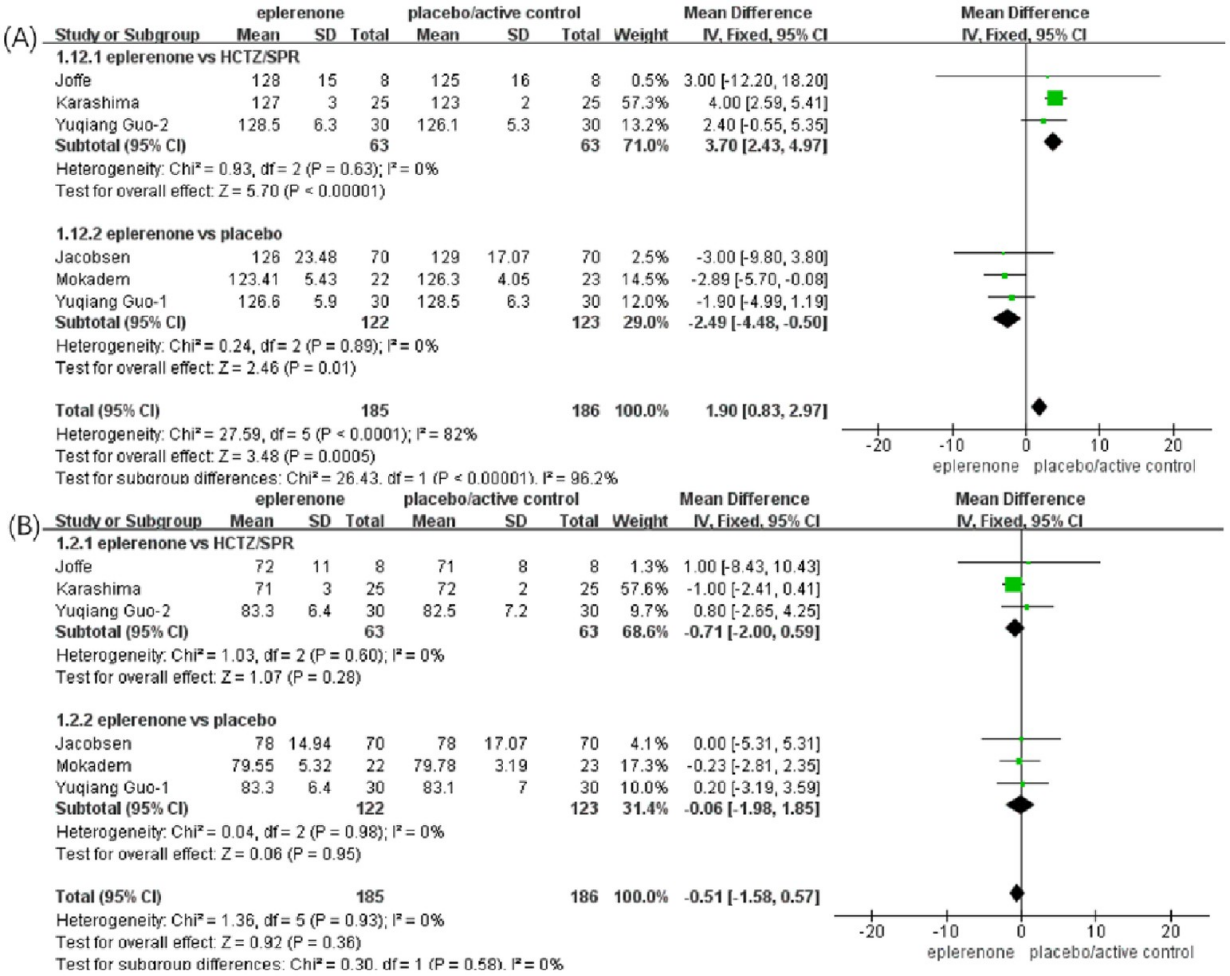
Forest plots of the effects between the eplerenone treatment groups and the controls. (A) Effect on SBP in articles comparing eplerenone to placebo/active control. (B) Effect on DBP in articles comparing eplerenone to placebo/active control.

In three articles comparing eplerenone to HCTZ/SPR, a significant change in SBP was indicated in the control group (MD, 3.70 [95% CI, 2.43 to 4.97], *P* = 0.0001), whereas in other three articles comparing eplerenone to placebo, there was a significant decrease in the eplerenone treatment group (MD, -2.49 [95% CI, -4.48 to -0.50], *P* = 0.01). In a pooled analysis of all articles, due to the different settings of the control subgroups, the heterogeneity in this analysis among subgroups was significantly different (*Chi*^*2*^ = 26.43, *P* < 0.0001, *I*^*2*^ = 96.2%) (Fig 5A).

However, compared with the controls in the pooled analysis of all articles, the eplerenone treatment groups displayed no significant difference in the change in DBP (MD, -0.51[95% CI, -1.58 to 0.57], *P* = 0.36), and we found no heterogeneity in this analysis among subgroups (*Chi*^*2*^ = 0.30, *P* = 0.58, *I*^*2*^ = 0%). In three articles comparing eplerenone to HCTZ/SPR, no obvious change in DBP levels was indicated (MD, -0.71 [95% CI, -2.00 to 0.59], *P* = 0.28), and in other three articles comparing eplerenone to placebo, no significant effect was identified either (MD, -0.06 [95% CI, -0.18 to 1.85], *P* = 0.95). (Fig 5B).

#### Effect of eplerenone treatment on LN

**Fig 6:**
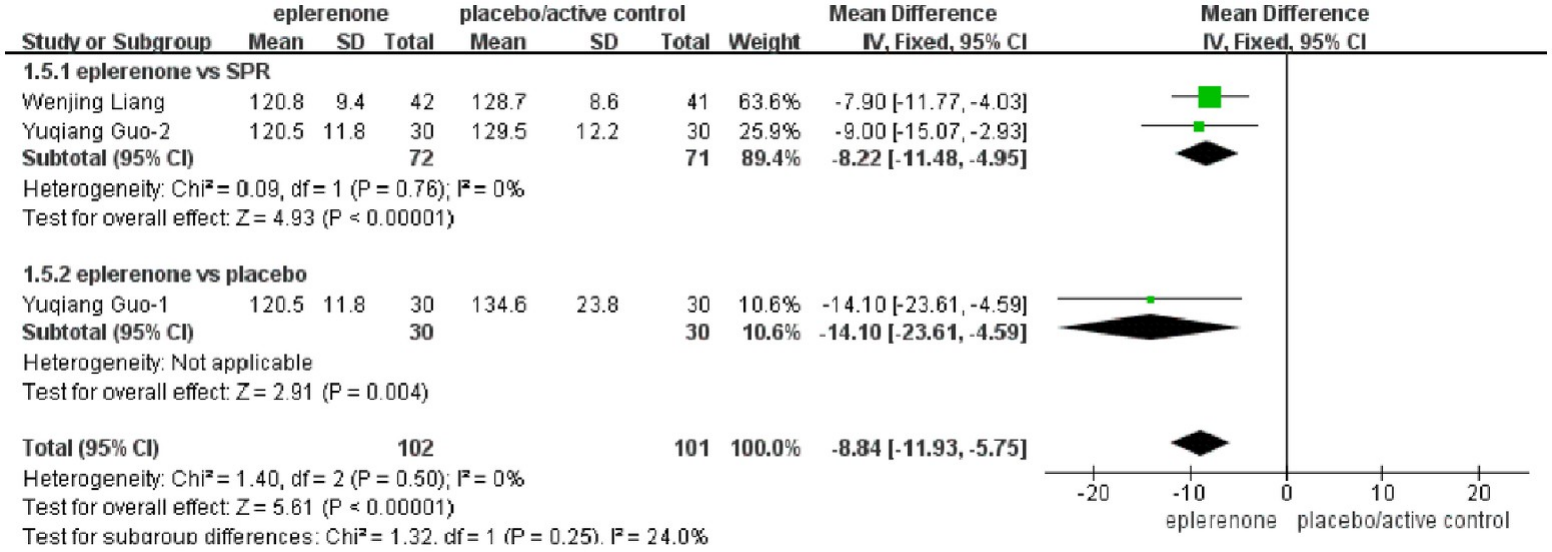
Forest plots of the effects on LN between the eplerenone treatment groups and the placebo/active controls.

Three articles (n = 706) indicated the effect of eplerenone on LN between the eplerenone treatment groups and the controls, and no significant heterogeneity was identified in the selected articles between the two subgroups (*Chi*^*2*^ = 1.32, *P* = 0.25, *I*^*2*^ = 24%). There was a reduction in LN in the eplerenone treatment group compared with the SPR controls (MD, -8.22 [95% CI, -11.48 to -4.95], *P* = 0.00001), and an obvious greater reduction in LN in the eplerenone treatment group than in the placebo controls (MD, -14.10 [95% CI, 123.61 to -4.59], *P* = 0.004). In the pooled analysis of all articles, the total effect was that LN level reduced significantly with eplerenone treatment, which was more effective than SPR treatment (MD, -8.84 [95% CI, -11.93 to -5.75], *P* = 0.00001) (Fig 6).

### Adverse events

#### Hyperkalemia

**Fig 7:**
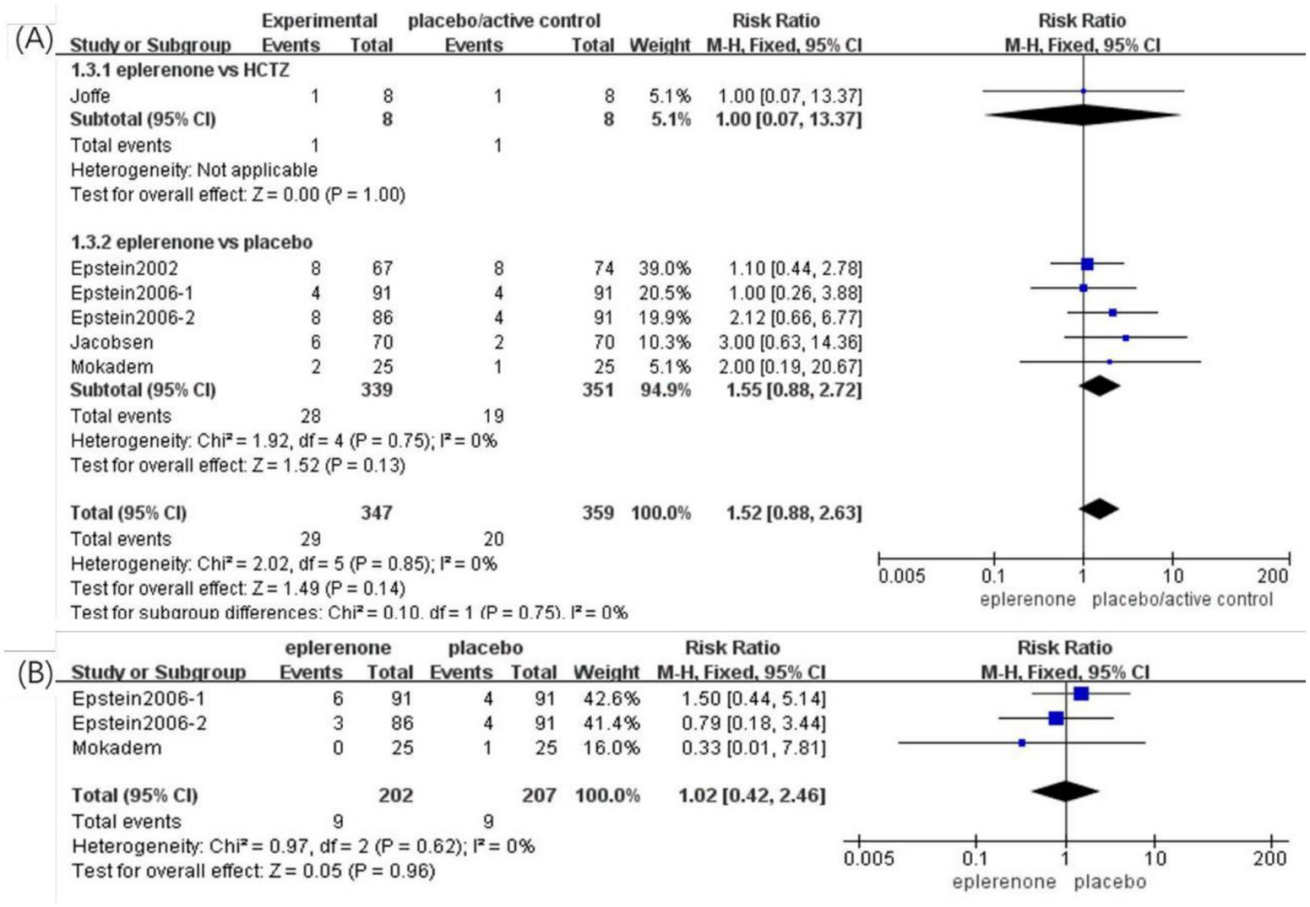
Forest plots of the effects between the eplerenone treatment groups and the controls. (A) Incidence of hyperkalemia in articles comparing eplerenone to placebo/active control. (B) Incidence of the adverse events of cough in articles comparing eplerenone to placebo control. The black diamond represents summary data centred on the pooled estimates with the risk ratio, and the width spans the corresponding 95% CIs.

Six articles (n = 706) indicated a difference in the relative risk of hyperkalemia between the eplerenone treatment groups and the controls. No significant difference was identified in the incidence of hyperkalemia between the eplerenone treatment groups and the controls (RR, 1.52 [95% CI, 0.88 to 2.72], *P* = 0.13). No significant heterogeneity was identified in the subgroup trials included here (*Chi*^*2*^ = 0.10, *P* = 0.75, *I*^*2*^ = 0%) (Fig 7A).

#### Cough

There were three articles (n = 409) that showed no obvious difference in the adverse events of cough between the two groups (RR, 1.02 [95% CI, 0.42 to 2.46], *P* = 0.96) (Fig 7B). No significant heterogeneity was identified between the articles analyzed (*Chi*^*2*^ = 0.97, *P* = 0.62, *I*^*2*^ = 0%).

### Assessment of sensitivity analysis and publication bias

**Fig 8:**
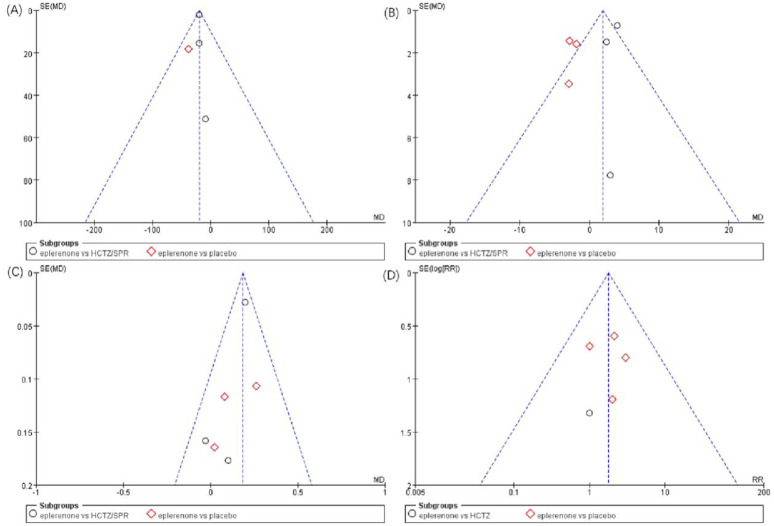
Funnel plots. Funnel plots of the effects of eplerenone treatment on (A) 24-h proteinuria (B) systolic pressure (C) potassium levels (D) adverse events of hyperkalemia at the end of follow-up.

As indicated by the sensitivity analysis of the selected articles, no article significantly interfered with the meta-analysis results, which meant that the selected articles had good stability. The funnel plots were systematically performed with the effectiveness and adverse event indicators, including 24-h proteinuria, systolic pressure, potassium levels, and hyperkalemia events, as shown in Fig 8(A) to 8(D). As indicated in Fig 8, the distributions of the (A), (C), and (D) funnel plots were symmetrical, and the scatter points of the study were within the scope of the funnel plots, demonstrating that the possibility of publication bias was not noticeable. Publication bias in Fig 8(B) was attributed to the different controls, whereas no significant heterogeneity was identified among the subgroup trials.

## Discussion

The development of DN in patients with T2DM is mainly driven by a combination of factors, including hemodynamic changes, metabolic disorders (e.g., blood lipids and blood glucose), and inflammatory/fibrotic factors [20]. Under the combined action of these factors, the body develops tubulointerstitial damage and pathological changes (e.g., inflammation, glomerulosclerosis, and glomerular hypertrophy), thereby causing renal fibrosis and ultimately the gradual progression of DN. Currently, clinical treatment mainly focuses on improving lifestyle, controlling hemodynamics (e.g., antihypertensive treatment), and improving metabolic disorders (e.g., glucose-lowering therapy and lipid-lowering therapy); the renin-angiotensin-aldosterone system is critical to the development of DN. As indicated in recent articles, excessive activation of the mineralocorticoid receptor (MR) acts as a vital link in causing renal, cardiac, and vascular lesions, and excessive activation of MR upregulates the expression of pro-inflammatory and profibrotic factors, thereby causing tubulointerstitial inflammation and fibrosis [21, 22]. As a second-generation aldosterone receptor inhibitor, eplerenone can bind to MR, inhibiting cellular pro-inflammatory and profibrotic gene transcription [23], and suppressing inflammation and fibrosis, thereby mitigating renal, vascular, and cardiac injuries. Moreover, MRAs can significantly suppress MCP-1 expression, glomerular macrophage infiltration, and phenotypic activation (i.e., α-smooth muscle actin expression) in animal models, thereby indicating an anti-fibrosis effect [24]. Thus, this study systematically analyzed the efficacy and safety of eplerenone in DN to further validate the appropriate clinical treatment.

As revealed in previous studies, glomerular hemodynamic variations, glomerular basement membrane thickening, negatively charged proteoglycan reduction, and podocyte and endothelial cell function variations are the vital pathogenesis of proteinuria in patients with diabetes. In addition, with the progression of the disease and glomerular morphological variations, especially podocyte damage and loss, glomerular endothelial cell injury will be further aggravated. This pathological process has a vicious cycle and accelerates the progression of DN. Furthermore, decreased tubular reabsorption protein capacity is a vital factor in proteinuria production [25]. The level of change in proteinuria, a monitoring indicator for the onset and progression of DN, predicts the risk of renal and cardiovascular disease progression in patients with T2DM [26]. In this meta-analysis, eplerenone treatment significantly improved 24-h urinary protein, microalbuminuria, and UACR levels in patients with DN compared with the controls, which is consistent with the results of other studies [27]. Studies have shown that the positive effect of eplerenone on proteinuria is achieved by affecting podocytes and maintaining the glomerular filtration barrier, which can significantly mitigate podocyte injury and regression and delay the progression of proteinuria and glomerulosclerosis even if the antihypertensive effect is not significant [16]. In this meta-analysis, because some articles did not provide complete data, only three articles were analyzed in the UACR. Accordingly, further study data are needed to assess the accuracy of the validation results. For eGFR, another important indicator of renal function, the decrease in eGFR is an essential predictor of renal endpoint events and is also closely correlated with cardiovascular events and death in patients with DN [26]. This meta-analysis identified no significant change in glomerular filtration rate with eplerenone treatment compared with the control, which is consistent with previous studies [5, 28]. Besides, from one level of consideration, there seems to be no apparent questioning factor in maintaining a relatively stable GFR in patients with DN with the combination of mineralocorticoid receptor antagonists based on current treatment. However, some studies have suggested that after treatment with eplerenone, a small proportion of patients showed deteriorated changes in renal function [29]. This suggests that eplerenone may be correlated with different baseline renal function in various study subjects; therefore, the renal function needs to be monitored in time during clinical application.

Hypertension is a crucial factor that induces diabetic microangiopathy, and patients with diabetes and hypertension have a much faster onset time of renal disease than those without hypertension, which can continue to worsen and lead to further deterioration of DN [30]. In this meta-analysis, the SBP-lowering effect of eplerenone was better than that of the placebo group but weaker than that of the HCTZ/SPR group, and DBP was not significantly different from that of the control. This result was not consistent with the significant antihypertensive effect of eplerenone reported in some studies [28, 31]. In addition, a study on refractory hypertension (HTN) [32] highlighted that eplerenone was effectively used to treat patients with mild to moderate HTN, and was an effective antihypertensive drug, whether used alone or in combination with other drugs. Its antihypertensive effect was identical in black and white patients, and even superior to that of ARB in black patients. It has also been stressed [29] that the antihypertensive mechanism of action of eplerenone mitigates renal sodium reabsorption by antagonizing aldosterone levels. In contrast, MRs are expressed in all cardiovascular tissues (e.g., vascular endothelial cells and smooth muscle cells) to reduce blood pressure by blocking signal transduction effects by MRAs. The following differences were identified in the results of this meta-analysis and many articles: 1. Experimental control drug factors. Hydrochlorothiazide inhibits the reabsorption of sodium chloride by the distal renal tubules and exerts a direct antihypertensive effect, and its antihypertensive effect may be more robust than that of MRAs, targeting only the weak diuretic effect of the distal renal tubules and collecting ducts. 2. Background drugs have been accompanied with ACEI/ARB in previous studies. This meta-analysis was partially combined, considering that there may exist differences. 3. Baseline differences in blood pressure control among the articles. The lack of significant difference in diastolic blood pressure reduction between this meta-analysis and the control may be correlated with mild hypertension or reasonable blood pressure control in some patients. 4. Limitations on the number and quality of the articles analyzed.

Renal fibrosis plays a vital role in DN development. Laminin, a non-collagen glycoprotein in the extracellular matrix, is widely distributed in the transparent layer of the basement membrane, abuts the cell base, participates in the growth and differentiation of cells, and is an indicator of renal fibrosis [33]. In this meta-analysis, LN reduced significantly after treatment in the eplerenone group, thereby demonstrating that renal fibrosis improved significantly in patients with diabetes, and also improved compared with the SPR control, demonstrating the anti-fibrotic effect of eplerenone. Animal studies have demonstrated that the degree of blockade of glomerular fibrosis can be determined by altering glomerular membrane expansion and reducing LN deposition. Eplerenone is a selective aldosterone receptor antagonist that selectively blocks mineralocorticoids and inhibits further oxidative stress and inflammatory injury, arterial remodeling, fibrosis, and kidney injury in DN [34]. Another animal model study suggested that eplerenone had no significant effect on body weight or blood pressure [35]. In contrast, the study suggested that eplerenone could significantly reduce oxidative stress, inhibit renal interstitial fibrosis, mitigate inflammatory responses manifested via macrophage and monocyte infiltration, and hinder the proliferation and activation of interstitial cells. However, the anti-renal fibrosis effect of eplerenone was not suggested to be correlated with its effect on blood pressure, similar to the results of this meta-analysis.

For drug side effects, the articles by Yuqiang Guo [14] and Wenjing Liang [15] suggested that some patients included in the study had occasional gastrointestinal discomfort, which was relieved after treatment. The Mokadem [17] study suggested that discontinuations due to side effects of the drug were infrequent in the respective treatment groups (one persistent hyperkalemia, one persistent dry cough, and one symptomatic hypotension). The remaining articles only mentioned changes in serum potassium levels. Owing to the factors of test data, this meta-analysis only performed statistical analysis on the change in serum potassium level, the risk of hyperkalemia, drug-induced dry cough, and other side effects, of which the side effect of drug-induced dry cough did not reveal significant differences compared with the control. In addition, given the pharmacological mechanism of drugs with MRAs, increased sodium excretion reduces renal potassium excretion, leading to increased serum potassium levels. In the conclusion of this meta-analysis, treatment with eplerenone significantly increased serum potassium levels compared with the control treatment.

In contrast, no significant difference in the risk of hyperkalemia was identified, similar to the results of some studies [36]. A previous study [37] showed that the risk of hyperkalemia being correlated with mineralocorticoid antagonists was 54%, while the risk of hyperkalemia not being correlated with MRA was 46%. However, some relevant articles have also pointed out that mineralocorticoid antagonism therapy may correlate with a significant risk of hyperkalemia [28, 29, 31].

On the one hand, relevant articles have confirmed that the concomitant use of ACEI/ARB drugs in hyperkalemia risk is correlated with an increased risk of hyperkalemia. In addition, it may be correlated with the differences in the baseline serum potassium level and baseline renal function in the respective study population analyzed. Hyperkalemia risk increases significantly in patients with high baseline serum potassium levels and poor renal function [38]. Furthermore, the respective studies had different threshold definitions for the risk of hyperkalemia, some defined it as ≥ 5.5 mmol/L, and others as 6.0 mmol/L.

In brief, this meta-analysis suggests that eplerenone can effectively reduce urinary protein and anti-fibrosis in patients with DN, and it also shows a specific improvement in blood pressure. Although it increases the blood potassium level to some extent, it does not significantly increase the risk of hyperkalemia. Eplerenone can act as an effective supplement to the existing clinical treatment for patients with DN.

This meta-analysis followed the evidence-based medicine research results to combine and analyze the collected clinical RCT results of eplerenone for treating DN, which addressed the defects of a small single experimental sample and inconsistent results, improved the statistical power, and presented more reliable evidence for clinical practice and decision-making. However, there were also the following limitations: (1) For the quality of the selected articles, the number of articles used in this meta-analysis was small, the sample size of some articles was small, and the data of some articles were insufficient. (2) Some articles were unblinded or single-blinded, and only 50% mentioned allocation concealment. (3) Most of the articles used in this analysis had a short follow-up time, and only one study had a planned follow-up of 46 weeks, which could not assess the efficacy and safety in the long term. (4) Given the data collection, no analysis was performed separately for the study population treated with ACEI drugs. (5) Owing to the factors of study data, only one article [16] studied sex hormone levels before and after treatment, therefore, there was no sufficient data to target hormone levels for statistical analysis to compare the side effects of different MRAs on sex hormones. Taken together, more high-quality, large-sample randomized controlled clinical studies should be used in future studies to clarify precisely the efficacy and safety of eplerenone treatment in patients with DN.

## Supporting information

S1 Checklist (DOCX)
